# Supplementary material for: Bone Marrow-Derived Mesenchymal Stromal Cells (MSCs) Modulate the Inflammatory Character of Alveolar Macrophages from Sarcoidosis Patients
Source: J Clin Med. 2020 Jan 19;9(1):278. doi: 10.3390/jcm9010278 (PMC7019909; doi:10.3390/jcm9010278)
Supplement: Supplementary file 1 [file jcm-09-00278-s001.pdf]

Suppl. Fig 1.

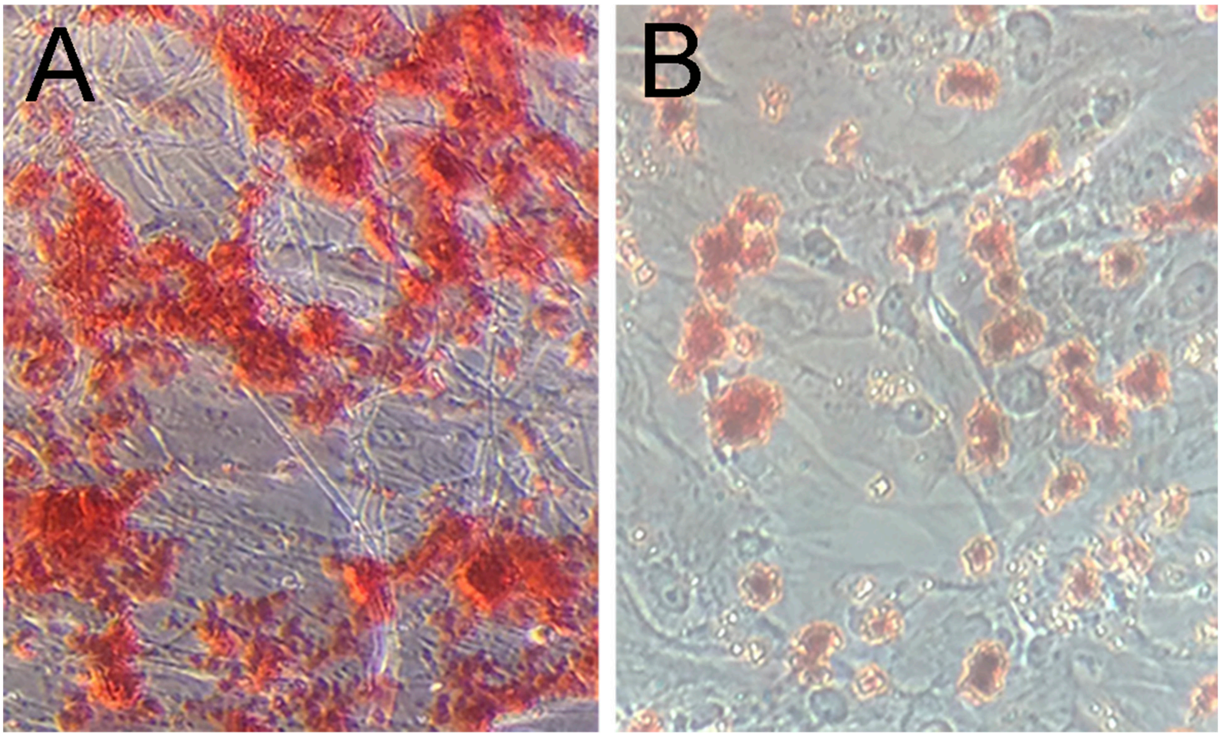

Suppl Fig 1. In vitro stains to demonstrate bi-lineage mesodermal differentiation potential of MSCs. Alizarin Red staining demonstrates osteogenic differentiation (A), Oil Red staining shows evidence of adipogenic differentiation (B).

Suppl. Fig. 2.

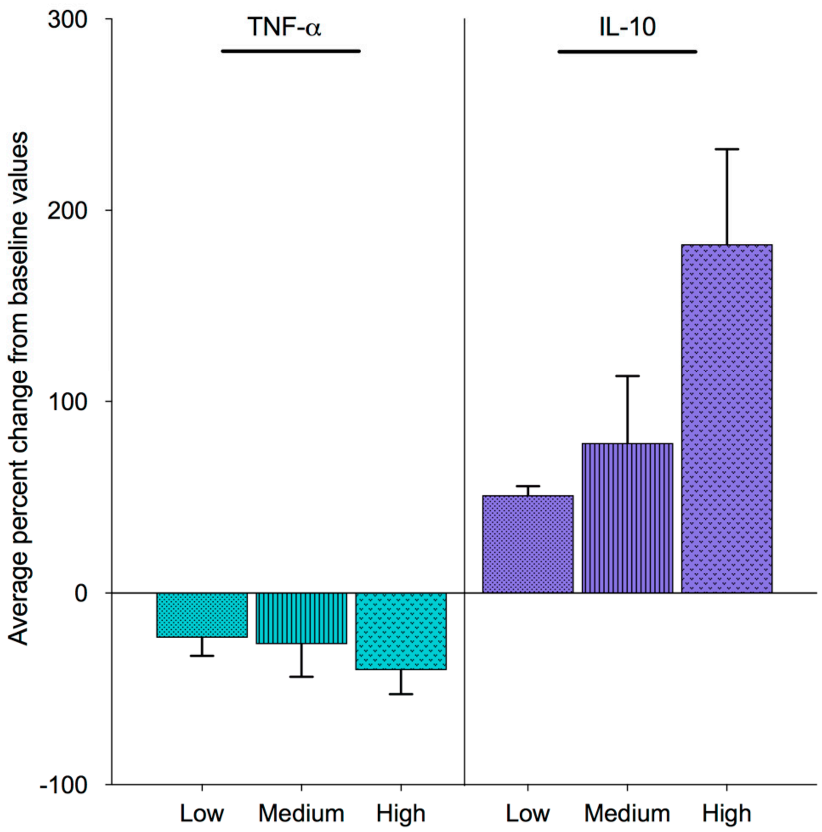

Suppl. Fig.2. Average percent change in sarcoidosis subject bronchoalveolar lavage (BAL) cell cytokine production across different bone marrow stromal cell (MSC) doses. Related to Figure 1. MSC/BAL cell ratio dependent immunomodulatory effects by MSCs were observed in 5 sarcoidosis samples tested. The aggregate data are presented in 3 different MSC/BAL cell ratios (low below 1:4, medium between 2:3 and 1:4, and high 1:1 and above). We chose a ratio (1 MSC to 10 BAL cells) that was efficient and reproducible in immune suppression effect and had optimal cell density for growth (n = 5; data are shown as mean ± SEM).

Suppl. Fig.3.

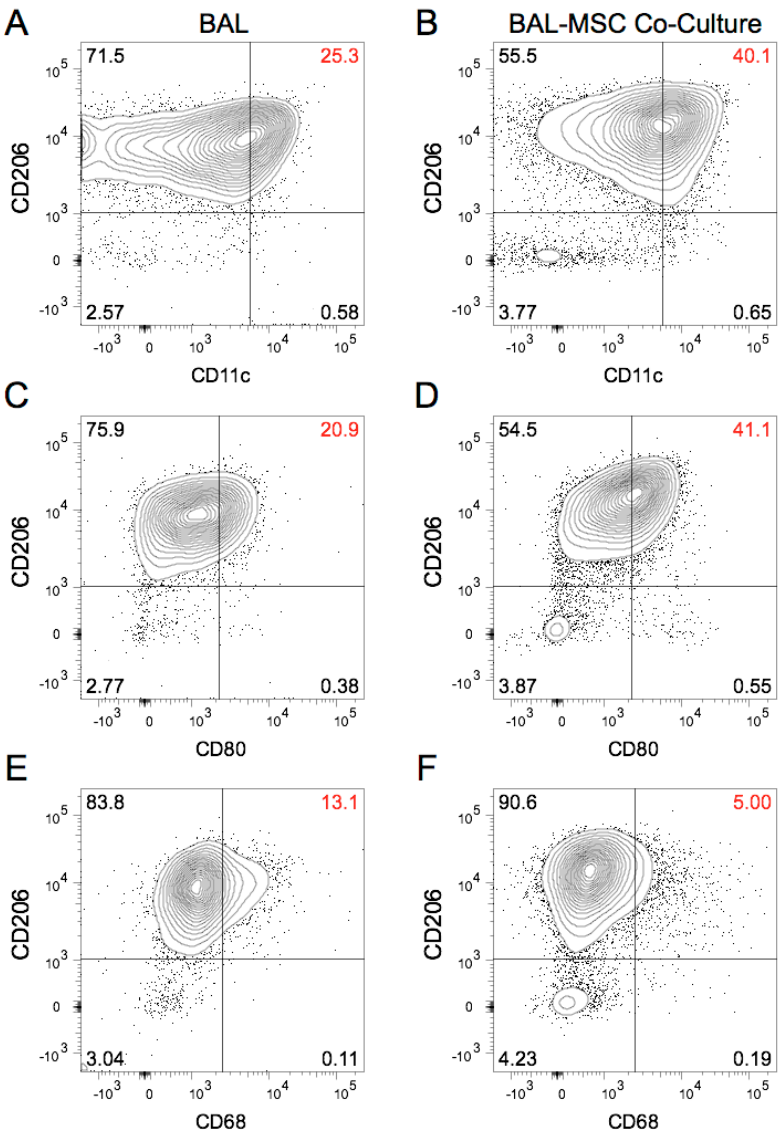

Suppl. Fig.3. Flow cytometry analysis of mononuclear cells (90% of which were macrophages) freshly prepared from a sarcoidosis subject (sarcoidosis subject 16). Related to Figure 2. Bronchoalveolar lavage (BAL) cells were plated and cultured for 16 hours (overnight) with (B, D, and F) and without (A, C, and E) the presence of bone marrow stromal cells (MSCs). Surface marker CD206 was used to identify alveolar macrophages (AMs) and flow cytometry was performed to determine changes in their surface marker expression. Co-cultured BAL cells

increased their expression of M1 associated markers CD11c (A and B) and CD80 (C and D), while decreasing expression of the macrophage marker CD68 (E and F).

Suppl. Fig.

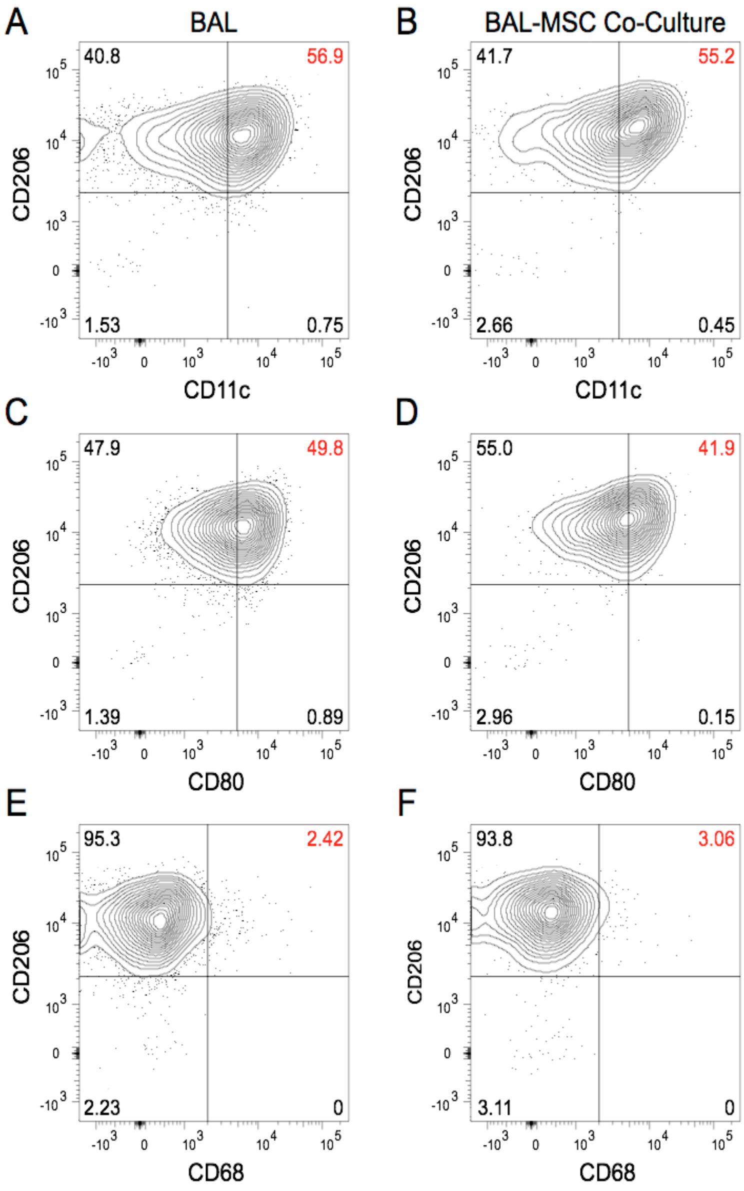

Suppl. Fig.4. Flow cytometry analysis of mononuclear cells (73% of which were macrophages) freshly prepared from a sarcoidosis subject (sarcoidosis subject 17). Related to Figure 4.

Bronchoalveolar lavage (BAL) cells were plated and cultured for 16 hours (overnight) with (B, D, and F) and without (A, C, and E) the presence of bone marrow stromal cells (MSCs). Surface marker CD206 was used to identify alveolar macrophages (AMs) and flow cytometry was performed to determine changes in their surface marker expression. Co-cultured BAL cells decreased their expression of M1 associated markers CD11c (A and B) and CD80 (C and D), while increasing expression of the macrophage marker CD68 (E and F).

Suppl. Table 1: Functional Results

Parameter	Controls (n = 7)	Sarcoidosis (n = 15)	P-Value
	**Mean Value**	**Mean Value**	
**Pulmonary Function Tests**			
FVC pre (L), (SD)	4.04 (0.50)	2.98 (1.02)	0.018
FVC pre % predicted (%), (SD)	97.86 (13.23)	84.6 (19.04)	NS
FEV1 pre (L), (SD)	3.23 (0.49)	2.16 (0.75)	0.0009
FEV1 pre % predicted (%), (SD)	97.14 (16.75)	78.7 (22.49)	0.05
FEV1/FVC pre, (SD)	79.7 (4.07)	73.3 (11.46)	0.07
TLC (L), (SD)	5.49 (0.66)	4.29 (1.21)	0.024
TLC % predicted (%), (SD)	93.7 (11.59)	83 (16.80)	NS
RV/TLC, (SD)	26 (4.80)	30.2 (7.36)	NS
DLCO adj (mL/mmHg/min), (SD)	25.6 (5.11)	17.45 (7.54)	0.009
DLCO adj % predicted (%), (SD)	74.57 (8.34)	59 (19.44)	0.016
**Six Minute Walk Test**			
Walk Distance (m), (SD)	547.9 (77.08)	474.9 (75.07)	0.05
SpO2, Post-test (%), (SD)	98.7 (1.11)	95.2 (5.41)	0.03
Borg Dyspnea Scale post-test, (SD)	2.1 (1.25)	3 (0.91)	NS

Abbreviations: Forced Vital Capacity pre-bronchodilator (FVC pre) ; Forced Expiratory Volume in 1 second, pre-bronchodilator (FEV1 pre); Total Lung Capacity (TLC); Diffusion Capacity adjusted for hemoglobin (DLCO adj); Residual Volume (RV); Pulse Oximetry (SpO2).

Parameter	Controls		Sarcoidosis		P-Value
	N	Mean Value	N	Mean Value	
Complete Blood Count (peripheral)					
WBC (K/mL), (SD)	8	5.43 (1.41)	15	6.22 (2.32)	NS
Lymphocyte count (K/mL), (SD)	8	2.07 (0.72)	15	1.74 (0.94)	NS
Lymphocytes (%), (SD)	8	38.85 (10.57)	15	27.60 (9.77)	NS
Monocyte count (K/mL), (SD)	8	0.37 (0.1)	15	0.60 (0.22)	0.011
Monocyte (%), (SD)	8	6.89 (1.12)	15	9.84 (2.64)	0.001
Platelet (K/mL), (SD)	8	246.9 (65.13)	15	269.34 (82.78)	NS
MPV (fL), (SD)	8	10.44 (0.44)	15	10.29 (0.72)	NS
Hemoglobin (g/dl), (SD)	8	13.37 (1.06)	15	13.23 (1.05)	NS
RDW (%), (SD)	8	13.86 (1.98)	15	13.64 (13.65)	NS
Chemistry					
Serum CRP-HS (mg/L), (SD)	8	4.66 (4.02)	15	5.05 (4.33)	NS
Serum ACE (U/L), (SD)	8	23.6 (15.46)	14	57.4 (37.6)	0.008

Suppl. Table 2: Laboratory Results

Abbreviations: C-reactive protein- high sensitivity (CRP-HS); Serum Angiotensin Converting Enzyme (serum ACE)

Suppl. Table 3: Peripheral Blood Lymphocyte Phenotype

Parameter	Controls (n=8)	Sarcoidosis (n= 14)	P-Value
	Mean Value	Mean Value	
CD3 (cells/ mL), (SD)	1468 (493.3)	1169 (688.3)	NS
CD3 (%), (SD)	73.54 (6.55)	68.16 (12.84)	NS
CD4/CD3 (cells/ mL), (SD)	978.6 (473.2)	728.9 (481.1)	NS
CD4/CD3 (%), (SD)	47.21 (11.44)	40.75 (8.32)	NS
CD8/CD3 (cells/ mL), (SD)	443.5 (169.6)	411.8 (268.7)	NS
CD8/CD3 (%), (SD)	23.35 (9.67)	25.57 (12.11)	NS
CD19 (cells/ mL), (SD)	265 (134.1)	321.4 (296.9)	NS
CD19 (%), (SD)	13.44 (4.01)	16.69 (8.37)	NS
NK (cells/ mL), (SD)	235.6 (120.2)	196.9 (112)	NS
NK (%), (SD)	12.04 (4.59)	14.73 (10.06)	NS
CD4/CD8 ratio, (SD)	2.4 : 1 (1.07)	2.0 : 1 (1.17)	NS

Suppl. Table 4: Summary of clinical data of two additional Sarcoidosis Patients

	Sarcoidosis patient 16	Sarcoidosis patient 17
**Gender**	F	F
**Age**	52	49
**BMI (m/kg^2)**	39.0	45.6
**Resp Meds**	Inhaled steroid/LABA, LAMA, SABA	Inhaled steroid/LABA, LAMA, SABA
**PFTs**		
FVC pre / (% pred)	2.40 / (71%)	2.33 / (87%)
FEV_1 pre / (% pred)	**1.06 / (40%)**	1.67 / (77%)
FEV_1/FVC	**44**	72
TLC / (% pred)	3.72 / (78%)	2.87 / (71%)
DLCO adj / (% pred)	16.5 / (63)	12.5 / (49%)
**Six minute walk test**		
Walk distance (m)	360	385
SpO2 Pre test (%)	98	93
SpO2 Post test (%)	**89**	93
Borg Fatigue Pre	**9**	2
Borg Fatigue Post	**10**	3
**CBC**		
WBC	6.18	3.92
Lymphocytes abs	1.15	0.95
Lymphocytes %	18.6	24.2
Monocytes abs	0.46	0.30
Monocytes %	7.4	7.7
**CXR stage**	III	III
**CXR description**	Lower lung predominant reticular markings and bibasilar interstitial markings; Potential perihilar consolidation in the right lower lobe; Prominence of the pulmonary vasculature	Many subcentimeter, bilateral radiodensities compatible with history of sarcoidosis; cardiothoracic ratio 13.5-14/28-28.5
**BAL segments**	(RUL, RML) mainly, and RLL	RUL and LUL
**Endobronchial findings**	Narrowing of multiple segmental airway orifaces.<br>Excessive Dynamic airway collapse of segmental airways	Narrowing of the LLL orifice; and narrowing the LUL anterior subsegments with excessive dynamic collapse. Narrowing/stenosis of the LLL basilar subsegment

Suppl. Table 5: Bronchoalveolar Lavage Cell Counts and Peripheral Blood Lymphocytes in two additional Sarcoidosis Patients

	Sarcoidosis Patient 16	Sarcoidosis Patient 17
**Bronchoalveolar Lavage Cell Counts**		
Total Cells (x 10^7)	32	10
Lymphocytes (%)	7	16
Macrophages (%)	90	73
**Peripheral Blood Lymphocytes, Flow Cytometry**		
CD3 (%)	71.2	45.1 *
CD3 (cells/mL)	819	428 *
CD4/CD3 (%)	59.5	32.3
CD4/CD3 (cells/ mL)	684	307
CD8/CD3 (%)	11.8	14
CD8/CD3 (cells/ mL)	136 *	133 *
CD19 (%)	13.9 ^^	34.2 ^^
CD19 (cells/mL)	160	325 ^^
NK Cell (%)	14.7	19.5
NK Cell (cells/mL)	169	185
CD4:CD8 ratio	5.03	2.3
